# SUN1 inhibits osteogenesis and promotes adipogenesis of human adipose‐derived stem cells by regulating α‐tubulin and CD36 expression

**DOI:** 10.1111/jcmm.70143

**Published:** 2024-10-09

**Authors:** Tingyu Fan, Jinhui Zhu, Wenqing Liu, Rongmei Qu, Asmat Ullah Khan, Yulian Shi, Jiaxuan Liu, Zhitao Zhou, Chujiang Xu, Jingxing Dai, Jun Ouyang

**Affiliations:** ^1^ Guangdong Provincial Key Laboratory of Digital Medicine and Biomechanics & Guangdong Engineering Research Center for Translation of Medical 3D Printing Application & National Virtual & Reality Experimental Education Center for Medical Morphology (Southern Medical University) & National Key Discipline of Human Anatomy, School of Basic Medical Sciences Southern Medical University Guangzhou China; ^2^ Central Laboratory Southern Medical University Guangzhou China; ^3^ Department of Orthopedics, TCM‐Integrated Hospital Southern Medical University Guangzhou China

**Keywords:** adipogenesis, CD36, osteogenesis, Sad1 and UNC84 domain 1, α‐Tubulin

## Abstract

Sad and UNC84 domain 1 (SUN1) is a kind of nuclear envelope protein with established involvement in cellular processes, including nuclear motility and meiosis. SUN1 plays an intriguing role in human adipose‐derived stem cells (hASCs) differentiation; however, this role remains largely undefined. This study was undertaken to investigate the role of SUN1 in hASCs differentiation, as well as its underlying mechanisms. Employing siRNAs, we selectively downregulated SUN1 and CD36 expression. Microtubules were depolymerized using nocodazole, and PPARγ was activated using rosiglitazone. Western blotting was performed to quantify SUN1, PPARγ, α‐tubulin, CD36, OPN, and adiponectin protein expression levels. Alkaline phosphatase and Oil red O staining were used to assess osteogenesis and adipogenesis, respectively. Downregulated SUN1 expression increased osteogenesis and decreased adipogenesis in hASCs, concomitant with upregulated α‐tubulin expression and downregulated CD36 expression, alongside reduced nuclear localization of PPARγ. Microtubule depolymerization increased CD36 expression. Rescue experiments indicated that microtubule depolymerization counteracted the downregulated SUN1‐induced phenotypic changes. This study demonstrates that SUN1 influences the differentiation of hASCs towards osteogenic and adipogenic lineages, indicating its essential role in cell fate.

## 1 INTRODUCTION

Regenerative medicine, underscored by its central emphasis on leveraging the potential of stem cells, is a revolutionary field in the realm of tissue repair and regeneration.^1^, ^2^ Within this framework, bone regeneration, in particular, has attracted attention, with the strategic application of stem cells emerging as a promising avenue to address injuries and disorders of bone tissue.^3^, ^4^, ^5^ Human adipose‐derived stem cells (hASCs) play a crucial role in bone regenerative medicine due to their ability to differentiate into osteoblasts.^6^, ^7^, ^8^, ^9^ Studies have shown that transplantation of hASCs in bone defect models significantly improves bone formation.^10^, ^11^, ^12^, ^13^ For example, transplanting hASCs in critical bone defects in mice has led to improved bone healing and structural integrity.^12^, ^14^ The osteogenic potential of hASCs can be modulated by regulation of key molecules, thus enhancing bone formation in vivo. For example, increased new bone formation has been reported upon transplantation of circ_0003204‐downregulated hASCs into a mouse bone defect model.^15^ Similarly, investigations involving hASCs with attenuated expression of AKR1C1 revealed an enhanced propensity for osteogenic differentiation.^16^ Therefore, efforts to elucidate strategies to promote osteogenesis in hASCs have become a focal point in contemporary research on bone tissue engineering.

Recent research has focused on nuclear envelope proteins because of their potential regulatory roles in cell functionality.^17^ Investigative efforts have supported a correlation between nuclear rheology and cell differentiation, highlighting the pivotal role of nucleoplasm/chromatin in determining the rheological characteristics of the cell nucleus.^18^ An intricate interaction between architecture and the number of nuclear pore complexes has been postulated, possibly leading to aberrant neurogenesis.^19^ Tang et al.,^20^ demonstrated the ability of the cell nucleus to adapt to mechanical stimuli by undergoing structural changes in the nuclear envelope. However, the mechanisms underlying the regulatory role of nuclear envelope proteins in the differentiation of hASCs remain unclear.

Our research endeavours have focused on elucidating the mechanisms of nuclear envelope molecules, particularly members of the Sad1 and UNC84 domain (SUN) family, in the differentiation of hASCs. The SUN family, primarily comprising Sad and UNC84 domain 1 (SUN1) and Sad and UNC84 domain 2 (SUN2), represents a group of proteins associated with the nuclear envelope.^21^, ^22^, ^23^ These proteins, through interactions with other proteins, establish connections between the nuclear membrane and the cellular cytoskeleton, playing a crucial role in the regulation of cell nucleus morphology and positioning, specifically during processes such as cell division and cell signalling.^24^, ^25^, ^26^, ^27^ The SUN family is recognized for its indispensable role in maintaining cell structure, regulating the cell cycle, and participating in various biological processes, including cell differentiation.^26^, ^27^, ^28^ Our previous study confirmed that SUN2 knockdown can affect microtubule reorganization and inhibit hASCs adipogenesis.^29^ However, the specific role of SUN1 in hASCs differentiation remains unknown.

Therefore, in this study, our aim was to explore the role of SUN1 in hASCs differentiation and to elucidate its underlying mechanistic intricacies. The findings of this study will contribute significantly to understanding the complex interactions between nuclear envelope proteins and the differentiation of hASCs, providing valuable information for the advancement of regenerative medicine.

## 2 MATERIALS AND METHODS

### 2.1 Cell differentiation

Human adipose tissue was obtained with informed consent from three donors by liposuction. The donors were healthy women aged 30–45 years. Adipose tissue was transferred to the centrifuge tube and 0.1% type I collagenase was added in equal volume. Digestion was performed on a 37°C shaker for 45 min, followed by an equal volume of growth medium to terminate digestion. Then centrifugation was performed at 1000 rpm at 4°C for 10 min. Cell pellets were suspended in growth medium and inoculated in the Petri dish, and incubated in an incubator at 37°C. The growth medium was changed every 2 days. Cells were passaged every 3–4 days and cells from passages 3–5 were used for the experiments.

Cells were seeded at a density of 2000/cm^2^ in 24‐well plates, 6‐well plates, or 6‐cm culture dishes. When reaching an approximate cell density of 80%, the osteogenic differentiation medium (10% fetal bovine serum [Gibco, USA], 37.5 mg/L ascorbic acid [Sigma, USA], 100 nM dexamethasone [Sigma, USA], 10 nM vitamin D3 [Solarbio, China], and 10 mM sodium glycerophosphate [Sigma, USA])^30^, ^31^ was replaced. The medium was changed every 2 days. Subsequently, after achieving a cell density of approximately 100%, the adipogenic differentiation medium (10% FBS, 0.1 nM 3‐isobutyl‐1‐methylxanthine [Sigma, USA], 1 μM dexamethasone, 10 g/mL insulin [Sigma, USA] and 100 μM indomethacin [Sigma, USA])^32^, ^33^, ^34^ was replaced. The medium was changed every 2 days.

The expression of key proteins was measured on Days 0, 1, 4, 7, 14 and 21 of differentiation. After a comparative analysis of osteogenic and adipogenic differentiation, we primarily focused on a 7‐day differentiation period, unless otherwise specified.

### 2.2 Western blotting assay

Whole protein lysates were prepared according to the manufacturer's instructions (KeyGEN, China). Subsequent to ice‐mediated cell lysis for 30 min, the cells were centrifuged at 12,000 rpm and 4°C for 15 min. The resulting supernatant was boiled with loading buffer for 5 min at 99°C. Subsequently, the samples were subjected to sodium dodecyl sulfate‐polyacrylamide gel electrophoresis, proteins were transferred onto a polyvinylidene fluoride membrane, and blocked with 5% skim milk for 1 h. Subsequently, primary antibodies were incubated at 4°C overnight, with antibody concentrations as follows: mouse anti‐OPN (ab69498, Abcam, Cambridge, UK, 1:1000); rabbit anti‐RUNX2 (ab114133, Abcam, Cambridge, UK, 1:1000); rabbit anti‐SUN1 (ab124770, Abcam, Cambridge, UK, 1:1000); β‐actin (ab8227, Abcam, Cambridge, UK, 1:2000); rabbit anti‐GAPDH (AP0063, Bioworld Technology, Minnesota, USA, 1:4000); rabbit anti‐PPARγ (MA5‐14889, Thermo Fisher Scientific, Massachusetts, USA, 1:1000); mouse anti‐CEBPβ (sc‐7962, Santa Cruz Biotechnology, Texas, USA, 1:700); mouse anti‐Adiponectin (ab22554, Abcam, Cambridge, UK, 1:1000); rabbit anti‐CD36 (ab252922, Abcam, Cambridge, UK, 1:4000); mouse anti‐α‐tubulin (ab7291, Abcam, Cambridge, UK, 1:2000). Secondary antibodies (HRP goat anti‐rabbit 1:5000, FuDe Biological Technology, Hangzhou, China; HRP goat anti‐mouse 1:5000, FuDe Biological Technology, Hangzhou, China) were incubated at room temperature for 1 h the following day. Band visualization was achieved by applying enhanced chemiluminescence (ECL) reagents (Fude Biological Technology, Hangzhou, China). Images were acquired using the Tanon chemiluminescence imaging system (Tanon5500 or Tanon5200, Shanghai Tianneng Life Science, China).

### 2.3 Immunofluorescence analysis

Cells were fixed with 4% paraformaldehyde (Solarbio, China) for 10 min, permeabilized with 0.1% Triton X100 (Solarbio, China) for 10 min, and blocked with 2% bovine serum albumin (Solarbio, China) for 1 h. The subsequent step involved an overnight incubation with primary antibodies at 4°C, with antibody concentration as follows: rabbit anti‐SUN1 (HPA008346, Atlas Antibodies, Stockholm, Sweden, 1:400) and rabbit anti‐PPARγ (MA5‐14889, Thermo Fisher Scientific, Massachusetts, United States, 1:1000). The samples were incubated with secondary antibodies at room temperature for 1 h the following day. The antibody concentrations and fluorescent dyes used were as follows: goat anti‐mouse‐568 (1:500, Beyotime Biotechnology, China), goat anti‐rabbit‐568 (1:500, Beyotime Biotechnology, China), goat anti‐mouse‐647 (1:500, Beyotime Biotechnology, China), goat anti‐rabbit‐647 (1:500, Beyotime Biotechnology, China), and phalloidin (1:500, Life Technologies, USA). Nuclei were stained with DAPI and images were acquired using a confocal microscope (Carl Zeiss LSM 880, Carl Zeiss AG, Oberkochen, Germany).

### 2.4 Alkaline phosphatase staining

The Alkaline phosphatase (ALP) working solution (Beyotime Biotechnology, China) was prepared in advance following the instructions. The samples were fixed with 4% paraformaldehyde for 10 min before being incubated with ALP working solution for 30 min.^30^, ^31^ Images were collected under an inverted microscope (Olympus 1MT‐2‐21, Olympus Corporation, Tokyo, Japan).

### 2.5 Alizarin red staining

Samples in a 24‐well plate were fixed with 4% paraformaldehyde for 10 min and stained with alizarin red S (ARS) (Cyagen, China) for 20–30 min.^30^, ^31^ Subsequently, images were acquired using an inverted microscope (Olympus 1MT‐2‐21, Olympus Corporation, Tokyo, Japan).

### 2.6 Oil red O staining

The oil red O working solution (Cyagen, China) was prepared following the manufacturer's instructions. After 10 min of fixation with 4% paraformaldehyde, the samples were subjected to incubation in oil red O working solution for 30 min,^29^ with subsequent image collection using an inverted microscope (Olympus 1MT‐2‐21, Olympus Corporation, Tokyo, Japan).

### 2.7 Cell transfection

Cells in passages 2–3 were used for transfection procedures, with lentiviruses obtained from GeneChem (Shanghai, China). Cell transfection was performed once the cell density reached 60%. An appropriate amount of lentiviral particles was added to the growth medium, and after incubating with the cells for 12 h, the medium was replaced with fresh growth medium. Following transfection, cells were expanded, incorporating selection with 0.1 μg/mL puromycin (GeneChem, China) for 2 days. The sequences for shSUN1 and shCD36 were CAGCGCAGAAGCACAAACAAA and GCCATAATCGACACATATAAA, respectively.

### 2.8 Quantitative real‐time polymerase chain reaction

Total RNA was extracted from cells using an RNA extraction kit (Dongsheng Biotech, China) and reverse‐transcribed using a RevertAid First Strand cDNA synthesis kit (Thermo Fisher, USA). The primer sequences used were as follows:

1. GAPDH‐F: AACAGCGACACCCACTCCTC;
2. GAPDH‐R: CATACCAGGAAATGAGCTTGACAA;
3. SUN1‐F: CTGTGAGACAGTGGATGCCGTA;
4. SUN1‐R: AGCATCGTCTGCAAGTCGCCTT.

### 2.9 Transwell assay

In the transwell assay, cells were suspended in serum‐free medium. The transwell chamber (Corning, USA) was placed in a 24‐well culture plate and a 200‐μL cell suspension was seeded in the upper chamber. After a 24‐h incubation period, cells in the upper chamber were removed, whereas cells in the lower chamber were fixed in 4% paraformaldehyde and stained with crystal violet. Image capture was performed using an inverted microscope.

### 2.10 Wound healing

For the wound healing assay, even horizontal lines were drawn across the bottom of a six‐well plate using a marker pen. Each horizontal line passed through the well at a spacing of approximately 1 cm. The cells were seeded in the wells and the cell density was adjusted according to the cell growth rates to cover the bottom of the well and to achieve a monolayer coverage after 24 h. A 10‐μL pipette tip was used to create wounds. The cells were then washed two to three times with sterile phosphate buffered saline and supplemented with fresh growth medium. The wound closure images were obtained at 0, 12 and 24 h post‐scratch. The experimental procedure was repeated three times, and Adobe Photoshop software (v. 2020) was used for image processing.

### 2.11 Microarray data analysis

The GSE75433 dataset^35^ (GSM1955098, GSM1955099, GSM1955100, GSM1955110, GSM1955111, GSM1955112, and GSM1955093) in the Gene Expression Omnibus database (https://www.ncbi.nlm.nih.gov/geo/) was analysed using GEO2R to obtain differentially expressed genes (DEGs) between osteogenesis and adipogenesis using the criteria of |logFC| > 0.5 and *p* < 0.05. The R software (R 3.6.0) was then used to generate a volcano map (ggplot2 package) and perform Gene Ontology (GO) and Kyoto Encyclopedia of Genes and Genomes (KEGG) enrichment analyses (ClusterProfiler package). Subsequently, a protein–protein interaction (PPI) network was constructed using the STRING database (https://cn.string‐db.org/).^36^ Lastly, key genes were identified using Cytoscape software (version_3.9.1) (CytoHubba).

### 2.12 Drug treatment

The concentrations used for rosiglitazone and nocodazole were 0.5 μM, and 100 ng/mL, respectively. The control group was supplemented with an equal amount of DMSO. The corresponding drug‐containing culture medium was replaced when the cell confluence reached approximately 80%.

### 2.13 Statistical analyses

All experiments were repeated a minimum of three times, and statistical data analysis was performed using GraphPad Prism v.7 software. Measurement data were represented as Mean ± SD. Differences between two and more groups were determined using the *t*‐test and one‐way ANOVA analysis, respectively. *p* < 0.05 represented statistical significance.

## 3 RESULTS

### 3.1 Differential expression of SUN1 in hASCs differentiation

**FIGURE 1:**
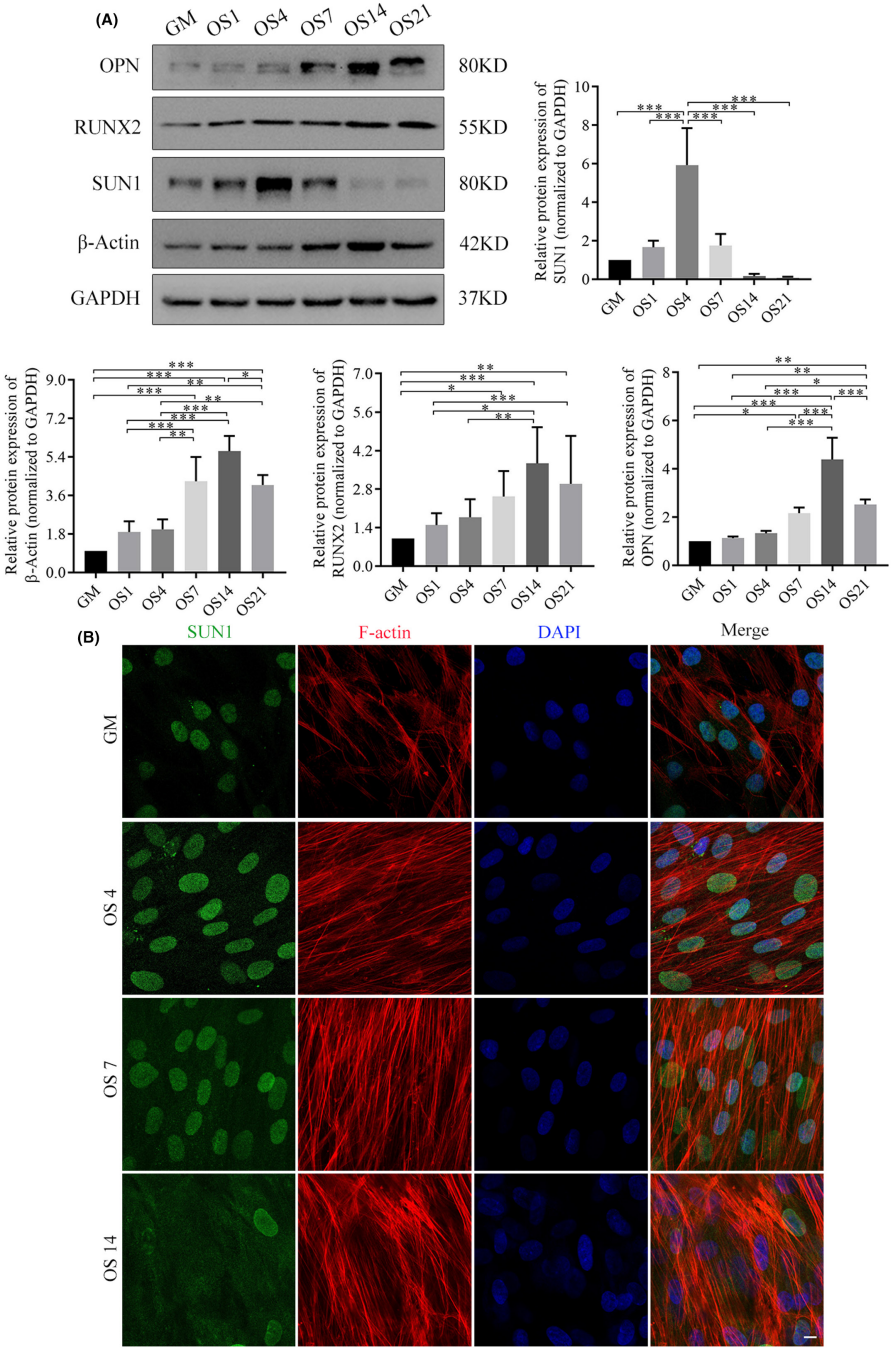
Differential expression of SUN1 in osteogenesis of hASCs. (A) The expression of SUN1, OPN, RUNX2, and β‐Actin in osteogenesis was detected by western blotting. GAPDH serves as an internal reference protein. *p < 0.05, **p < 0.01, ***p < 0.001. (B) The morphological changes of SUN1 in osteogenesis were detected by immunofluorescence assay. Scale bar, 10 μm. Green, SUN1; red, Actin filaments; and blue, the nuclei; GM, growth medium; OS, osteogenic differentiation medium. OS1, OS4, OS7, OS14, and OS21, representing Days 1, 4, 7, 14 and 21 of osteogenic differentiation, respectively.

To elucidate the role of SUN1 in hASCs differentiation, we conducted a bifurcated approach, exploring osteogenesis and adipogenesis, two interconnected processes with reciprocal influences.^37^, ^38^ Osteogenesis was induced in hASCs by applying an osteogenic differentiation medium and SUN1 expression was monitored during differentiation. On staining evaluations, ALP and ARS revealed a stronger stain intensity in the osteogenic differentiation medium (OS) group than in the growth medium (GM) group (Figure S1A–C). Western blotting (WB) assays indicated a gradual increase in the expression of the OPN and RUNX2 proteins, which serve as osteogenic markers. This increase peaked on Day 14 and exhibited a subsequent decline on Day 21. Interestingly, the β‐actin protein exhibited a similar trend. However, SUN1 protein expression reached its peak on Day 4, followed by a subsequent decline (Figure 1A and Figure S1D). Our previous study showed that Day 7 is a critical period in the osteogenesis process.^30^, ^31^ Based on these findings, we hypothesized that Day 7 could be a significant regulatory stage during which SUN1 may have a distinct impact on osteogenic and adipogenic differentiation. Therefore, we selected Day 7 as the time point for cell treatment. Immunofluorescence staining revealed that SUN1staining almost overlapped that of the nucleus (Figure 1B).

**FIGURE 2:**
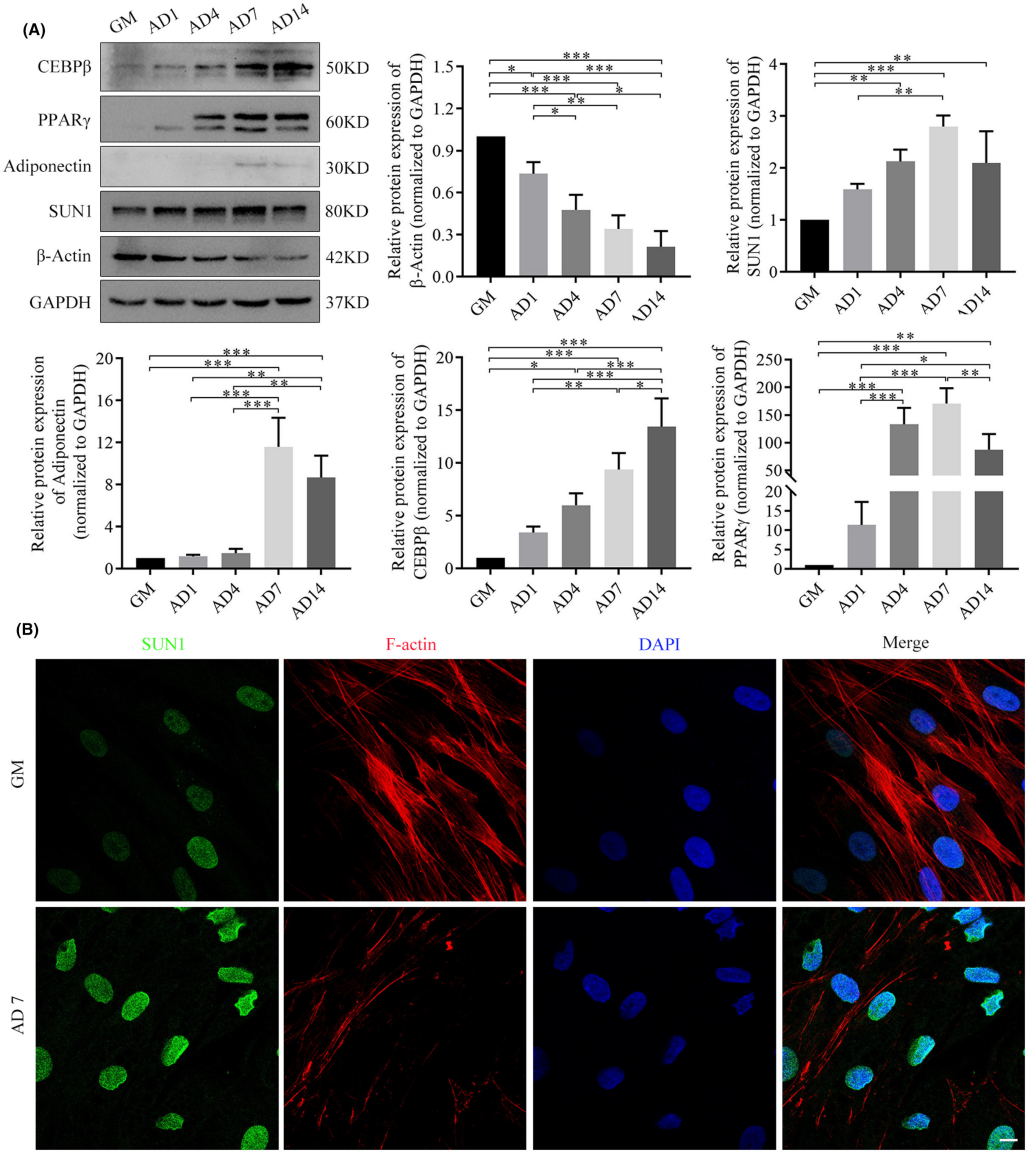
Differential expression of SUN1 in the adipogenesis of hASCs. (A) The expression of CEBPβ, PPARγ, adiponectin, SUN1, and β‐Actin in adipogenesis was detected by western blotting. GAPDH serves as an internal reference protein. *p < 0.05, **p < 0.01, ***p < 0.001. (B) The morphological changes of SUN1 in adipogenesis were detected by immunofluorescence assay. Scale bar, 10 μm. Green, SUN1; red, Actin filaments; and blue, the nuclei; GM, growth medium; AD, adipogenic differentiation medium. AD1, AD4, AD7, and AD14, representing Days 1, 4, 7 and 14 of adipogenic differentiation, respectively.

During adipogenesis, Oil red O staining revealed a gradual increase in the number of lipid droplets (Figure S2A,B). The subsequent WB assay indicated that compared to the uninduced group, the expression of adipogenesis markers, such as CEBPβ, PPARγ, and adiponectin, was upregulated during adipogenesis (Figure 2A). Compared with uninduced cells, SUN1 expression was upregulated during adipogenesis, reaching its peak expression on Day 7 (Figure 2A & Figure S2C). Furthermore, the protein expression of β‐actin decreased gradually during adipogenesis. Immunofluorescence staining revealed distinctive nuclear boundary invaginations on Day 7, with the SUN1 protein exhibiting analogous morphological changes. A notable decline in the number of actin fibres was observed, accompanied by a transformation in their morphology, characterized by curvature and shortened lengths (Figure 2B).

These results suggest that SUN1 expression was downregulated during the middle to late stages of osteogenesis and upregulated during adipogenesis in hASCs.

### 3.2 Effects of SUN1 downregulation on osteogenesis and adipogenesis of hASCs

**FIGURE 3:**
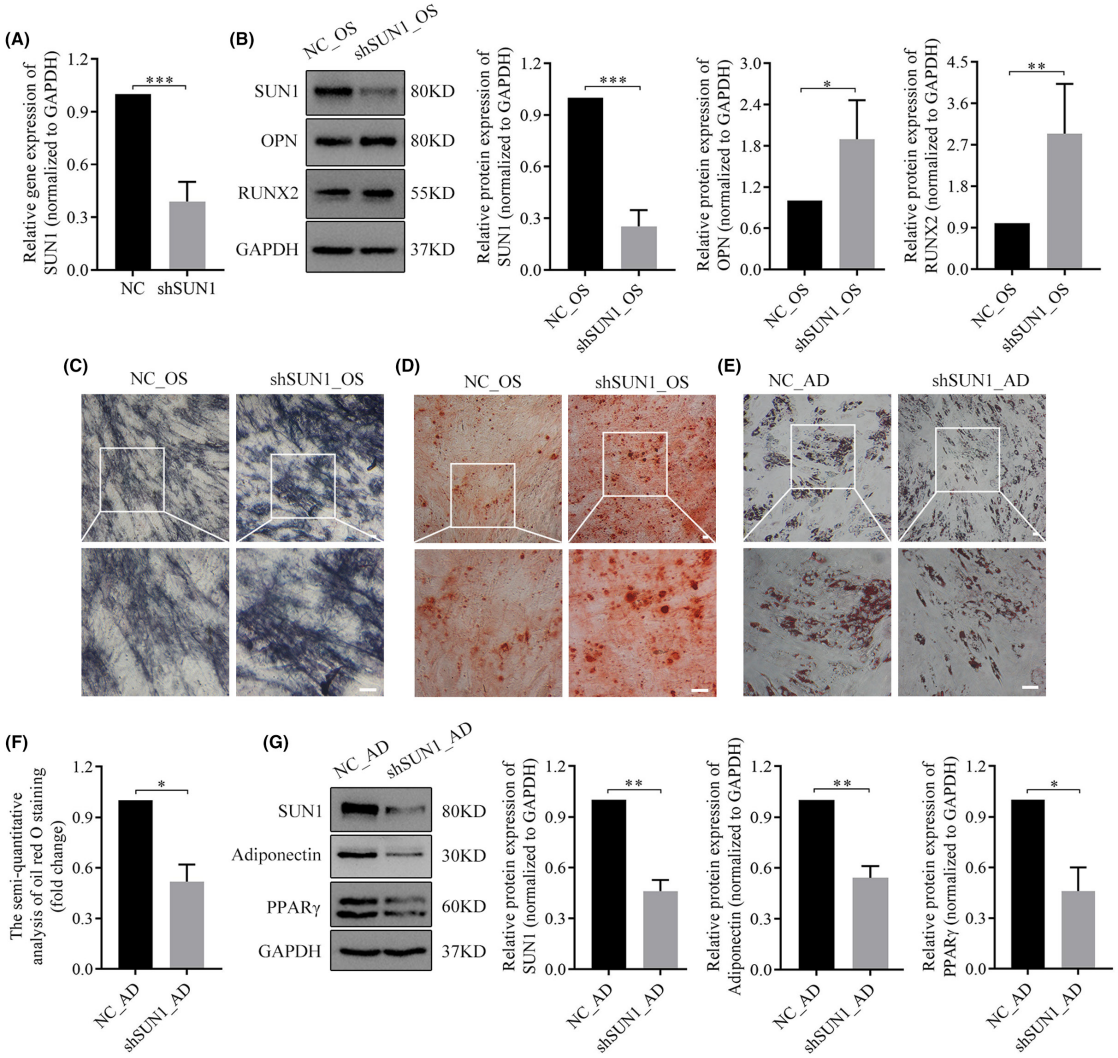
Effect of downregulated SUN1 on osteogenesis and adipogenesis of hASCs. (A) qRT‐PCR assay analysis of SUN1 gene expression. (B) The protein expression of SUN1, OPN, and RUNX2 was analysed by western blotting. GAPDH serves as an internal reference protein. ALP staining (C), and alizarin red staining (D) were used to identify osteogenesis. Scale bar, 50 μm. (E‐F) Oil red O staining. Scale bar, 50 μm. (G) The protein expression of SUN1, adiponectin, and PPARγ was analysed by western blotting assay. GAPDH serves as an internal reference protein. NC, negative control cells; shSUN1, SUN1 downregulated cells; OS, osteogenic differentiation medium; AD, adipogenic differentiation medium. *p < 0.05, **p < 0.01, ***p < 0.001.

To elucidate the role of SUN1 in hASCs differentiation, we employed siRNA to downregulate the expression of SUN1. Quantitative real‐time polymerase chain reaction (qRT‐PCR) results revealed approximately 60% downregulation in SUN1 mRNA expression in shSUN1 cells compared to the negative control group (Figure 3A). Furthermore, we observed a distinct alteration in cellular morphology, with shSUN1 cells assuming a slender morphology compared to cells in the negative control group (Figure S3A). The transwell assay results revealed an increased presence of cells in the lower compartment of the shSUN1 group relative to that of the negative control group after a 24 h period (Figure S3B). The wound healing assay revealed a faster healing rate in the shSUN1 group than in the negative control group (Figure S3C). These findings suggest a negative correlation between SUN1 expression and cell migration ability.

To clarify the effect of downregulation of SUN1 expression on osteogenesis, cells in both the negative control and shSUN1 groups were subjected to osteogenic differentiation. The WB assay revealed that, on Day 7, the shSUN1 group exhibited increased expression of OPN and RUNX2 proteins and SUN1 expression remained low during osteogenesis (Figure 3B). Subsequent evaluation by ALP and ARS staining assays revealed a stronger staining intensity in the shSUN1 group than in the negative control group (Figure 3C,D). These findings suggest that SUN1 downregulation leads to enhanced osteogenic differentiation in hASCs. Oil red O staining revealed a lower presence of lipid droplets in the shSUN1 group than in the negative control group (Figure 3E,F). WB analysis demonstrated that adiponectin and PPARγ expression levels in the shSUN1 group were approximately half of those of the negative control group (Figure 3G). These results indicate that with suppression of SUN1, adipogenesis decreases.

### 3.3 SUN1 negatively regulates α‐tubulin to inhibit osteogenesis and promote adipogenesis

**FIGURE 4:**
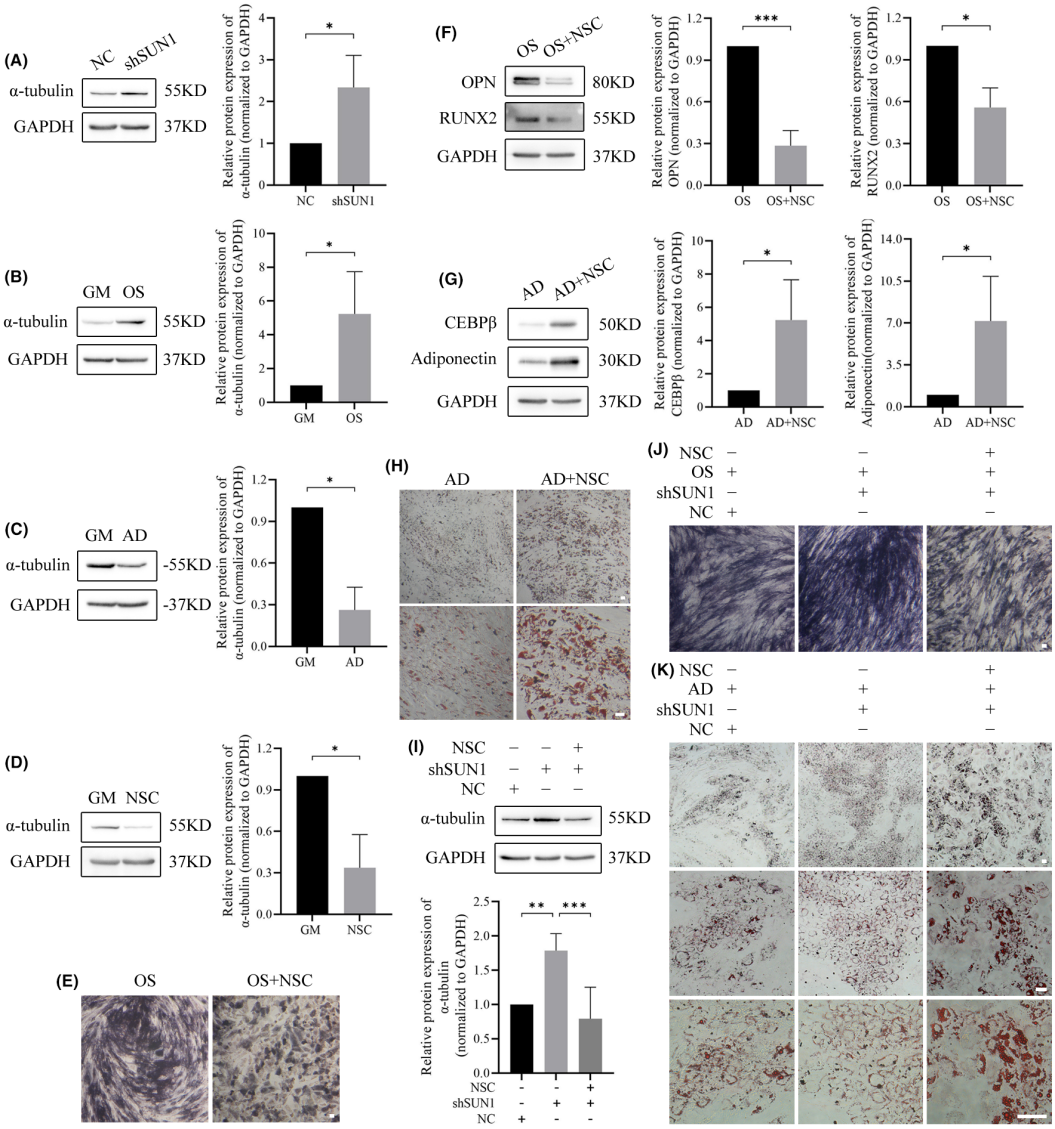
SUN1 negatively regulates α‐tubulin to inhibit osteogenesis and promote adipogenesis. (A–D) The protein expression of α‐tubulin was analysed by western blotting. (E) ALP staining. Scale bar, 50 μm. (F, G) The protein expression of OPN, RUNX2, CEBPβ, and adiponectin was analysed by western blotting. (H) Oil red O staining. Scale bar, 50 μm. (I) The protein expression of α‐tubulin was analysed by western blotting. (J) ALP staining. Scale bar, 50 μm. (K) Oil red O staining. Scale bar, 50 μm. GAPDH serves as an internal reference protein. NC, negative control cells; shSUN1, SUN1 downregulated cells; OS, osteogenic differentiation medium; AD, adipogenic differentiation medium; NSC, nocodazole. *p < 0.05, **p < 0.01, ***p < 0.001.

In light of our previous study revealing that cross‐talk between microtubules and SUN2 can regulate adipogenesis,^29^ we postulated a potential regulatory link between SUN1 and microtubules. To explore this hypothesis, we performed a WB assay to assess the expression of α‐tubulin, a prominent microtubule marker,^39^, ^40^, ^41^ in SUN1‐downregulated cells. Increased expression of α‐tubulin was observed in SUN1 downregulated cells (Figure 4A). Furthermore, the expression of α‐tubulin was upregulated and downregulated during osteogenesis and adipogenesis, respectively (Figure 4B,C). In particular, microtubule depolymerization using nocodazole (NSC) treatment resulted in a decrease in α‐tubulin levels (Figure 4D). Consequently, cells treated with osteogenic differentiation medium containing NSC exhibited decreased osteogenesis (Figure 4E,F). In contrast, adipogenesis was enhanced in cells treated with NSC‐containing adipogenic differentiation medium (Figure 4G,H). NSC demonstrated an ameliorative effect on the increase of α‐tubulin levels induced by downregulation of SUN1 (Figure 4I). In particular, rescue experiments further confirmed the regulatory role of SUN1 in modulating osteogenesis and adipogenesis. Treatment of SUN1 downregulated cells with osteogenic differentiation medium containing NSC resulted in a decrease in the intensity of ALP staining compared with that in the SUN1 downregulated group (Figure 4J). In contrast, treatment of SUN1 downregulated cells with adipogenic differentiation medium containing NSC resulted in an increase in lipid droplets compared with that of the SUN1 downregulated group (Figure 4K). These findings suggest that SUN1 can exert a dual regulatory effect, enhancing osteogenesis while suppressing adipogenesis, achieved by negatively regulating α‐tubulin expression.

### 3.4 CD36 serves as a downstream regulatory molecule influenced by SUN1 and α‐tubulin

**FIGURE 5:**
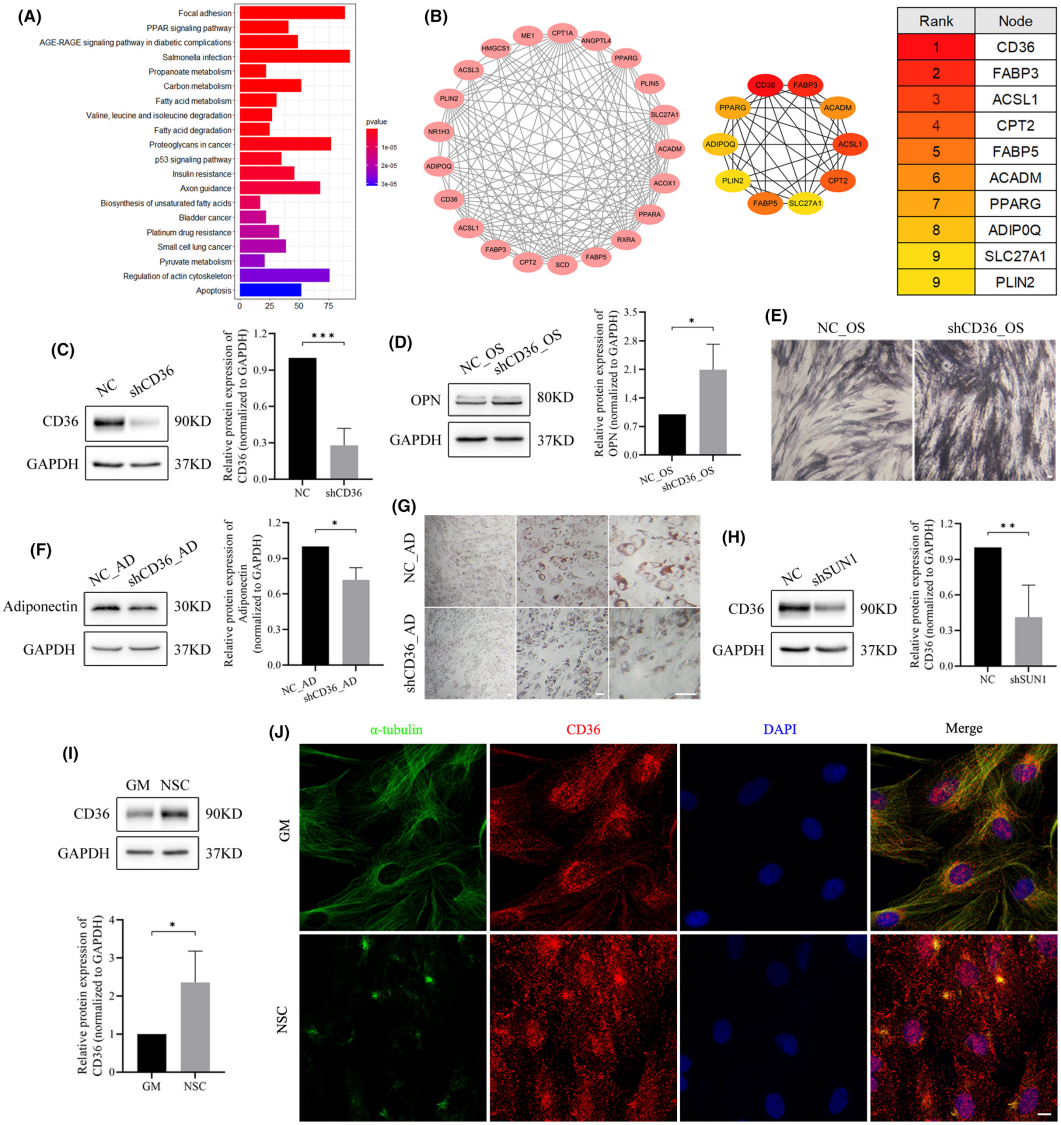
CD36 serves as a downstream regulatory molecule influenced by both SUN1 and α‐tubulin. (A) KEGG pathway analyses of differentially expressed genes (DEGs). (B) The PPI network of DEGs identified with Cytoscape software. (C, D) The protein expression of CD36 and OPN was analysed by western blotting. (E) ALP staining. Scale bar, 50 μm. (F) The protein expression of adiponectin was analysed by western blotting. (G) Oil red O staining. Scale bar, 50 μm. (H, I) The protein expression of CD36 were analysed by western blotting. (J) The co‐localization between microtubules and CD36 was observed by immunofluorescence assay. Scale bar, 10 μm. GAPDH serves as an internal reference protein. *p < 0.05, **p < 0.01, ***p < 0.001.

To identify key molecules associated with the regulatory role of SUN1 in the differentiation phenotype of hASCs, we performed a comparative analysis of DEGs between osteogenesis and adipogenesis in hASCs using the GSE75433 dataset. Through GO/KEGG enrichment and PPI network analyses, we identified the top 10 key genes, with CD36 ranking first based on gene scores (Figure 5A,B and Figure S4). To delineate the role of CD36 in cell differentiation, we used siRNA to downregulate its expression (Figure 5C). Subsequent investigations revealed enhanced ALP staining and increased expression of OPN during osteogenesis (Figure 5D,E). Simultaneously, during adipogenesis, a reduction in lipid droplet formation and a decrease in adiponectin expression were observed (Figure 5F,G). These results indicate a functional parallelism between CD36 and SUN1, with both exerting inhibitory effects on osteogenesis while simultaneously promoting adipogenesis. To verify whether CD36 is responsible for the downstream activity of SUN1 and α‐tubulin, we measured CD36 protein expression in SUN1‐downregulated cells. A decrease in CD36 expression was observed in SUN1 downregulated cells (Figure 5H), indicating that SUN1 positively regulated CD36 expression. When microtubule depolymerization was inducible, the expression of the CD36 protein was upregulated (Figure 5I). The immunofluorescence assay demonstrated colocalization of microtubule proteins with CD36; however, this colocalization decreased after microtubule depolymerization (Figure 5J), suggesting that microtubules negatively regulated CD36. These findings suggest that CD36 served as a downstream regulatory molecule influenced by both SUN1 and α‐tubulin.

### 3.5 SUN1 regulates cell differentiation by modulating PPARγ nuclear translocation

**FIGURE 6:**
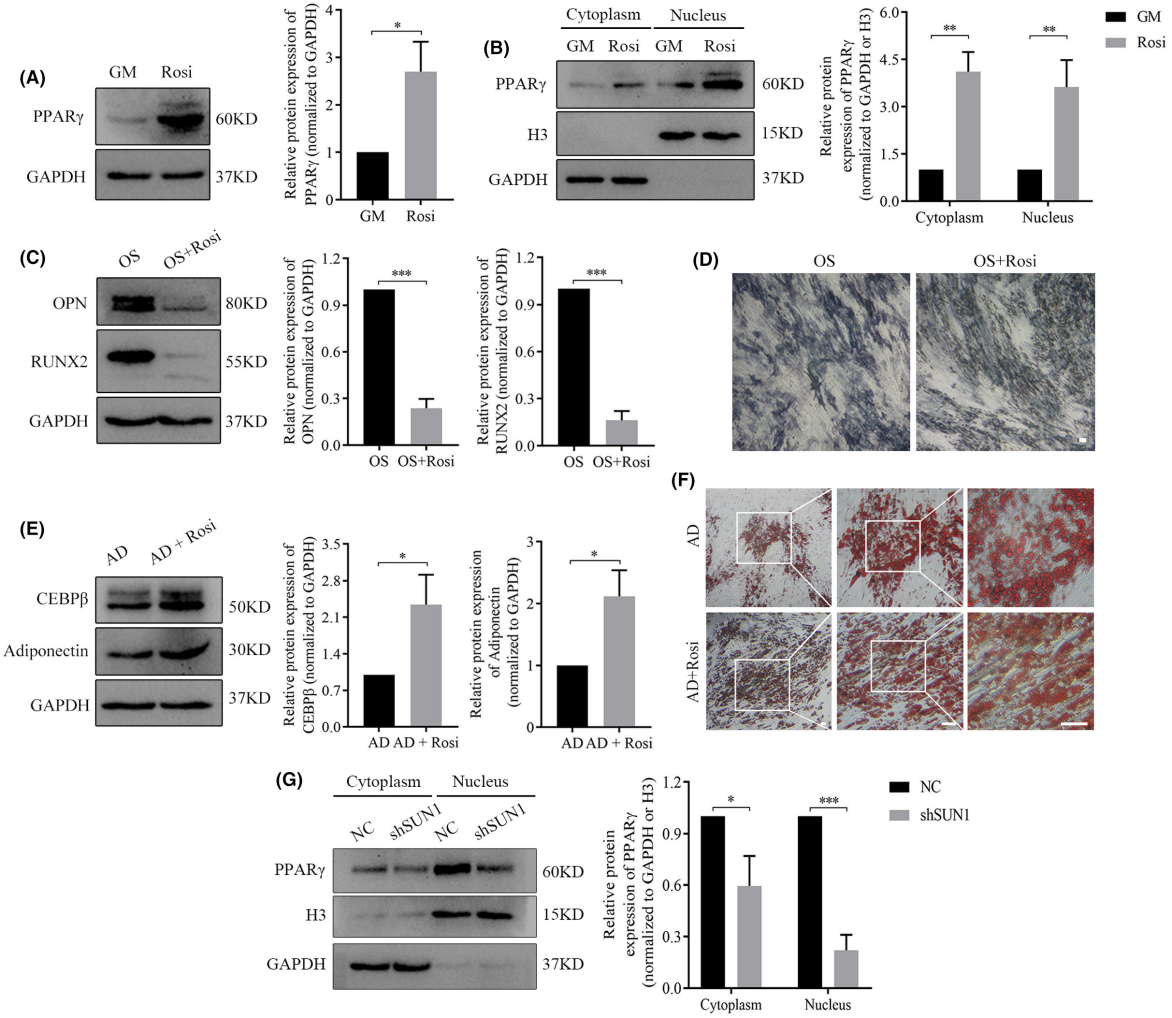
SUN1 positively regulated PPARγ nuclear translocation. (A–C) The protein expression of PPARγ, OPN, and RUNX2 was detected by western blotting. (D) ALP staining. Scale bar, 50 μm. (E) The protein expression of CEBPβ and adiponectin was detected by western blotting. (F) Oil red O staining. Scale bar, 50 μm. (G) The protein expression of PPARγ was analysed by western blotting. GAPDH serves as an internal reference protein. GM, growth medium; Rosi, rosiglitazone; OS, osteogenic differentiation medium; AD, adipogenic differentiation medium; NC, negative control cells; shSUN1, SUN1 downregulated cells. *p < 0.05, **p < 0.01, ***p < 0.001.

Bioinformatics analysis revealed that the PPARG signalling pathway was significantly enriched, with the PPARG gene ranked among the top 10 key genes (Figure 5A,B). Therefore, we hypothesized that PPARG, specifically PPARγ, similar to CD36, could be under the regulatory influence of SUN1. To evaluate the impact of PPARγ on cell differentiation potential, rosiglitazone (Rosi) was used as an activator. WB indicated a substantial increase in PPARγ protein expression after Rosi treatment (Figure 6A). Considering that the nucleus is the pivotal site for PPARγ function, we evaluated PPARγ protein in the cytoplasm and nucleus (Figure 6B). WB experiments revealed that OPN and RUNX2 protein expression were lower in the OS + Rosi group than in the OS group (Figure 6C). Moreover, ALP staining revealed a weaker intensity in the OS + Rosi group than that in the OS group (Figure 6D). The WB assay demonstrated that the AD + Rosi group exhibited an upregulation in CEBPβ and adiponectin expression (Figure 6E), complemented by an increased generation of lipid droplets (Figure 6F). These findings suggest that PPARγ exerted an inhibitory effect on osteogenesis while promoting adipogenesis in hASCs, reflecting the functional paradigm of the SUN1 protein. Moreover, PPARγ expression decreased both in the cytoplasm and in the nucleus in SUN1‐downregulated cells (Figure 6G). These results indicate that SUN1 can positively regulate PPARγ nuclear translocation, influencing cell differentiation.

**FIGURE 7:**
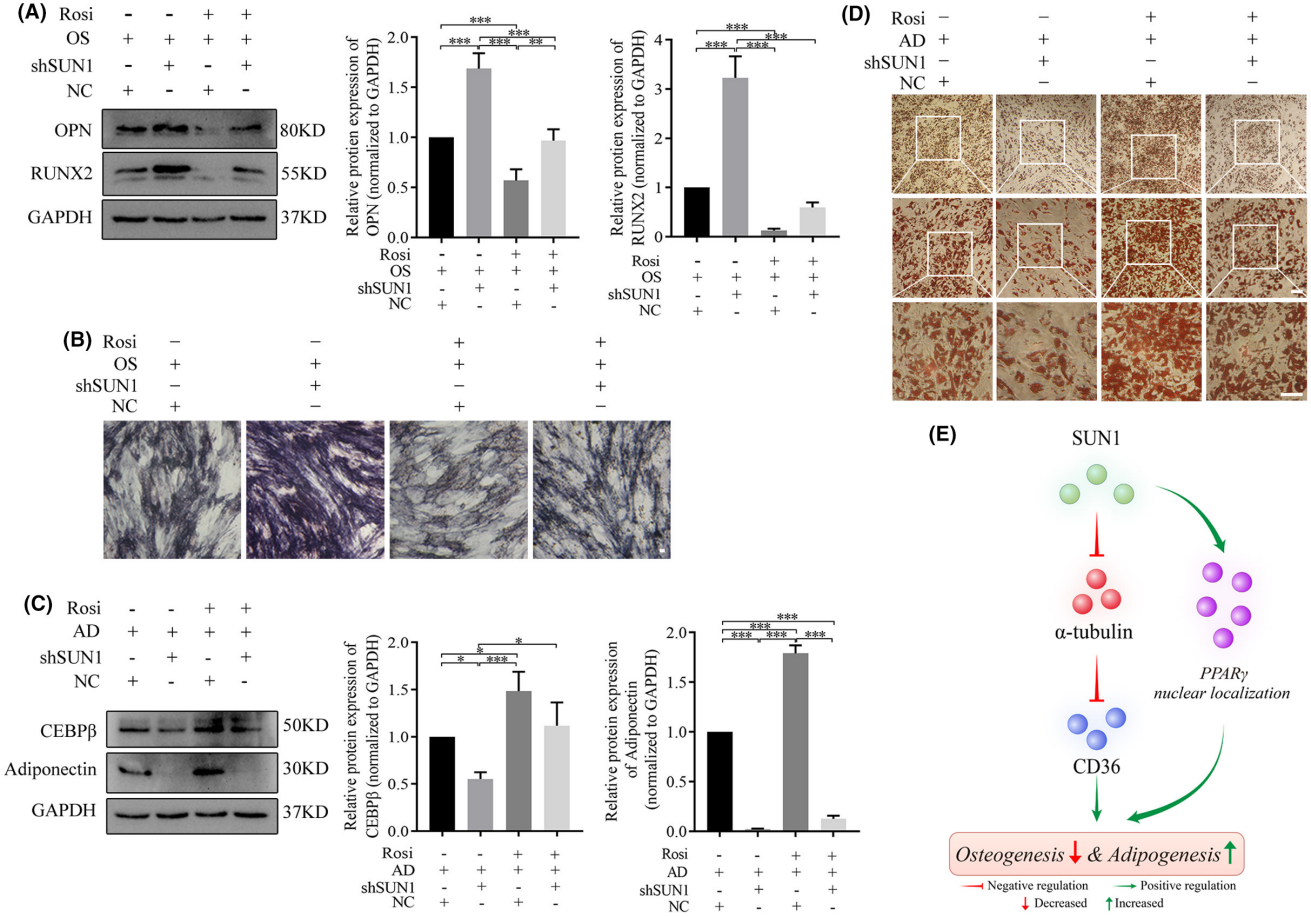
SUN1 regulates hASCs differentiation through PPARγ. (A) The protein expression of OPN and RUNX2 was detected by western blotting. (B) ALP staining. Scale bar, 50 μm. (C) The protein expression of CEBPβ and adiponectin was detected by western blotting. (D) Oil red O staining. Scale bar, 50 μm. GAPDH serves as an internal reference protein. OS, osteogenic differentiation medium; AD, adipogenic differentiation medium; Rosi, rosiglitazone; NC, negative control cells; shSUN1, SUN1 downregulated cells. *p < 0.05, **p < 0.01, ***p < 0.001. (E) Schematic diagram. SUN1 inhibited osteogenesis and promoted adipogenesis in hASCs by regulating α‐tubulin and CD36. Additionally, SUN1 modulated this differentiation phenotype by regulating the nuclear translocation of PPARγ.

Rescue experiments were carried out to determine the necessity of PPARγ in the regulatory framework of SUN1 during osteogenesis in hASCs. The results revealed that SUN1 downregulated cells treated with Rosi exhibited decreased expression of OPN and RUNX2 during osteogenesis compared to SUN1 downregulated cells without Rosi treatment (Figure 7A). This pattern was consistently mirrored in ALP staining (Figure 7B). Compared to Rosi‐treated cells, SUN1‐downregulated cells treated with Rosi exhibited decreased CEBPβ and adiponectin during adipogenesis (Figure 7C). Similarly, Oil‐red O staining exhibited analogous findings (Figure 7D). Altogether, these experimental results indicate that SUN1 inhibited osteogenesis and promoted adipogenesis by affecting α‐tubulin and CD36. In particular, the regulatory roles of SUN1 extended to the nuclear localization of PPARγ during cell differentiation (Figure 7E).

## 4 DISCUSSION

During the process of bone formation, the balance between osteogenesis and adipogenesis is crucial.^42^, ^43^ This balance maintains bone integrity and metabolic health, and when it is impaired, it can lead to diseases such as osteoporosis.^44^, ^45^ Given this interrelationship, our study focused on osteogenesis and adipogenesis as the main directions of differentiation of hASCs and explored the role of SUN1 in these processes.

SUN1 is a key component of the nuclear pore complex and plays a crucial role in nuclear positioning and morphology through its interaction with nuclear pore proteins.^46^ However, the specific effects of SUN1 on osteogenesis and adipogenesis in hASCs are not clear. By downregulating the expression of SUN1, we demonstrated for the first time the regulatory role of SUN1 in the differentiation of hASCs. Our experimental results show that downregulation of SUN1 expression led to increased osteogenesis and decreased adipogenesis in hASCs. Mechanistically, the downregulation of SUN1 led to increased expression of α‐tubulin and decreased expression of CD36, suggesting that SUN1 may influence cell fate by regulating the cytoskeleton and fat metabolism pathways. Rescue experiments further demonstrated that SUN1 regulated hASCs differentiation by affecting the nuclear localization of PPARγ. However, Ueda et al. showed that SUN1 knockdown can lead to instability of the microtubule system, triggering a series of protein reactions.^47^, ^48^, ^49^ This differs from our results, which we believe could be due to the use of different cell types: Our study used hASCs, while Ueda et al. used HeLa and MCF10A cell lines. This suggests that SUN1 may have different regulatory mechanisms in different cell types. Furthermore, our previous study showed that SUN2 knockdown increased microtubule expression and inhibited adipogenic differentiation in hASCs.^29^ This study serves as a continuation and complement to our previous research. Taken together, our findings and those of Ueda and others do not conflict, but rather highlight the diversity and complexity of the functions of the members of the SUN family.

In this study, we also found that adiponectin expression decreased in the SUN1 knockdown group during adipogenic differentiation, suggesting a possible link between the two in cell differentiation. As an important metabolic regulator, adiponectin modulates adipocyte differentiation through PPARγ and AMPK pathways,^50^, ^51^, ^52^ whereas SUN1 plays a role in nuclear structure and signal transduction.^46^ We hypothesize that SUN1 may influence cell differentiation by regulating adiponectin‐related signalling pathways. We plan to further explore the mechanisms of interaction between SUN1 and adiponectin in future studies to reveal their specific functions and potential applications in determining cell fate.

Human ASCs are derived from adipose tissue and possess the ability to differentiate into osteoblasts, offering a promising source of cells for bone regeneration.^11^, ^12^ When conventional treatments fail, hASCs can serve as an alternative source of stem cells, opening new avenues for therapeutic interventions. For example, hASCs can be transplanted into the bone marrow to replace reduced or impaired stem cells in patients with osteoporosis, thus helping to form new bone.^14^ With a deeper understanding of the differentiation mechanisms of hASCs, we can identify key factors that promote their osteogenic differentiation, thus developing new cell therapy strategies. For example, by regulating key factors such as SUN1, the transformation of hASCs into osteoblasts can be effectively enhanced, further improving bone density and mass. Thus, it is important to explore the mechanisms of differentiation of hASCs in the treatment of osteoporosis. However, our study still needs further confirmation of the clinical validity and safety of these findings in vivo. We plan to focus our future studies on exploring the use of hASCs in animal models and clinical trials to determine their actual effect on the treatment of osteoporosis.

Our findings not only deepen our understanding of the differentiation mechanism of hASCs, but also provides a new perspective and experimental basis for bone regeneration research.

## AUTHOR CONTRIBUTIONS

**Tingyu Fan:** Conceptualization (equal); data curation (equal); writing – original draft (equal); writing – review and editing (equal). **Jinhui Zhu:** Data curation (equal); software (equal); writing – original draft (equal). **Wenqing Liu:** Formal analysis (equal); resources (equal). **Rongmei Qu:** Data curation (equal); supervision (equal). **Asmat Ullah Khan:** Data curation (equal); investigation (equal). **Yulian Shi:** Investigation (equal); writing – original draft (equal). **Jiaxuan Liu:** Data curation (equal); methodology (equal). **Zhitao Zhou:** Data curation (equal); methodology (equal). **Chujiang Xu:** Funding acquisition (supporting); project administration (equal); supervision (equal). **Jingxing Dai:** Funding acquisition (equal); project administration (equal); supervision (equal); writing – review and editing (equal). **Jun Ouyang:** Funding acquisition (equal); project administration (equal); supervision (equal); writing – review and editing (equal).

## FUNDING INFORMATION

This study was financially supported by the National Key R&D Program of China (2022YFF1202603) and the President's Foundation of TCM‐Integrated Hospital of Southern Medical University (1202103001).

## CONFLICT OF INTEREST STATEMENT

The authors declare that they have no conflicts of interest regarding this work.

## CONSENT

Not applicable.

## Supporting information


Figure S1.

## Data Availability

All the supporting data can be downloaded.
