# Supplementary material for: SUN1 inhibits osteogenesis and promotes adipogenesis of human adipose‐derived stem cells by regulating α‐tubulin and CD36 expression
Source: J Cell Mol Med. 2024 Oct 9;28(19):e70143. doi: 10.1111/jcmm.70143 (PMC11463318; doi:10.1111/jcmm.70143)
Supplement: Supplementary file 1 — Figure S1. [file JCMM-28-e70143-s001.docx]

**Supplementary Figures**


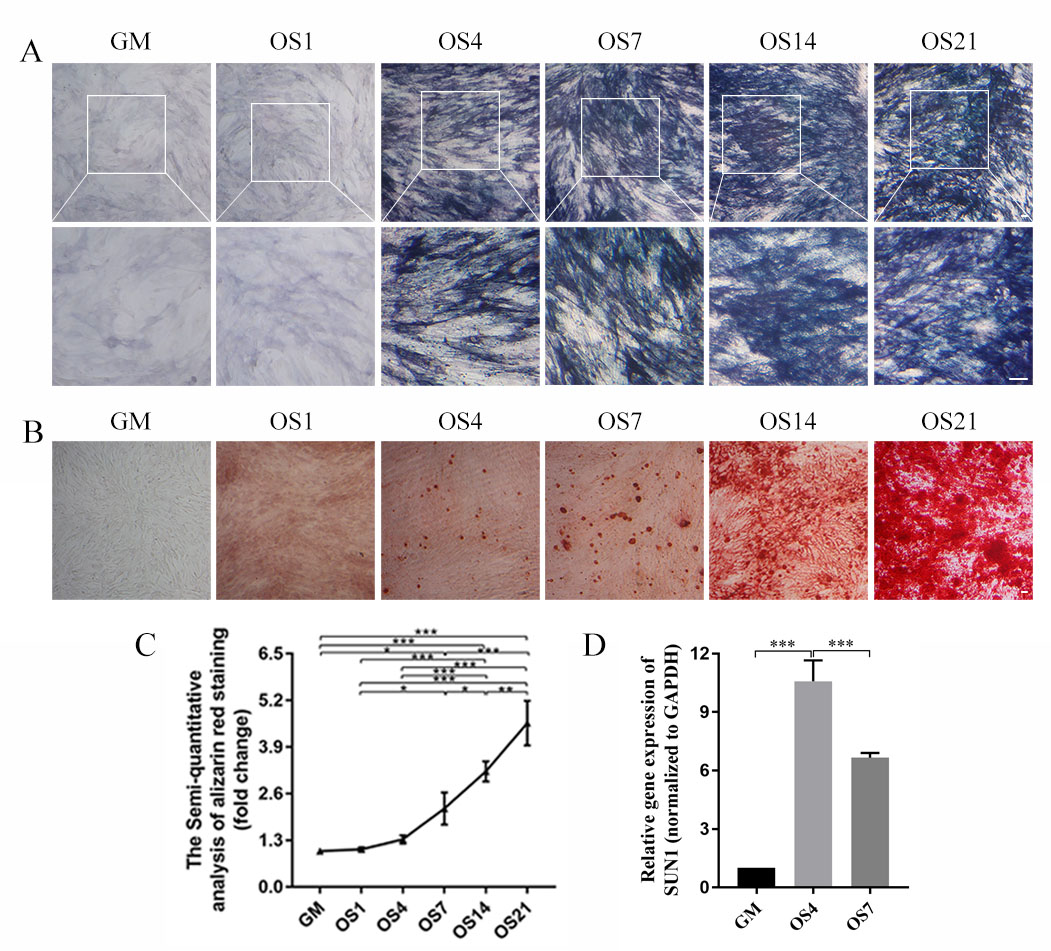


**Figure S1** **Identification of osteogenic differentiation of hASCs. (A)** ALP staining was performed to assess osteogenesis at different times. **(B-C)** Representative images of Alizarin Red staining and semi-quantitative analysis showing mineralized matrix formation at different times during osteogenic differentiation. **(D)** qRT-PCR assay analyzes the gene expression of SUN1. GM, growth medium; OS, osteogenic differentiation medium. OS1, OS4, OS7, OS14, and OS21 represent day 1, 4, 7, 14, and 21 of osteogenic differentiation, respectively. Scale bar, 50 μm. Experiments were repeated at least three times (n ≥ 3). **P* < 0.05, ***P* < 0.01, ****P* < 0.001.

**
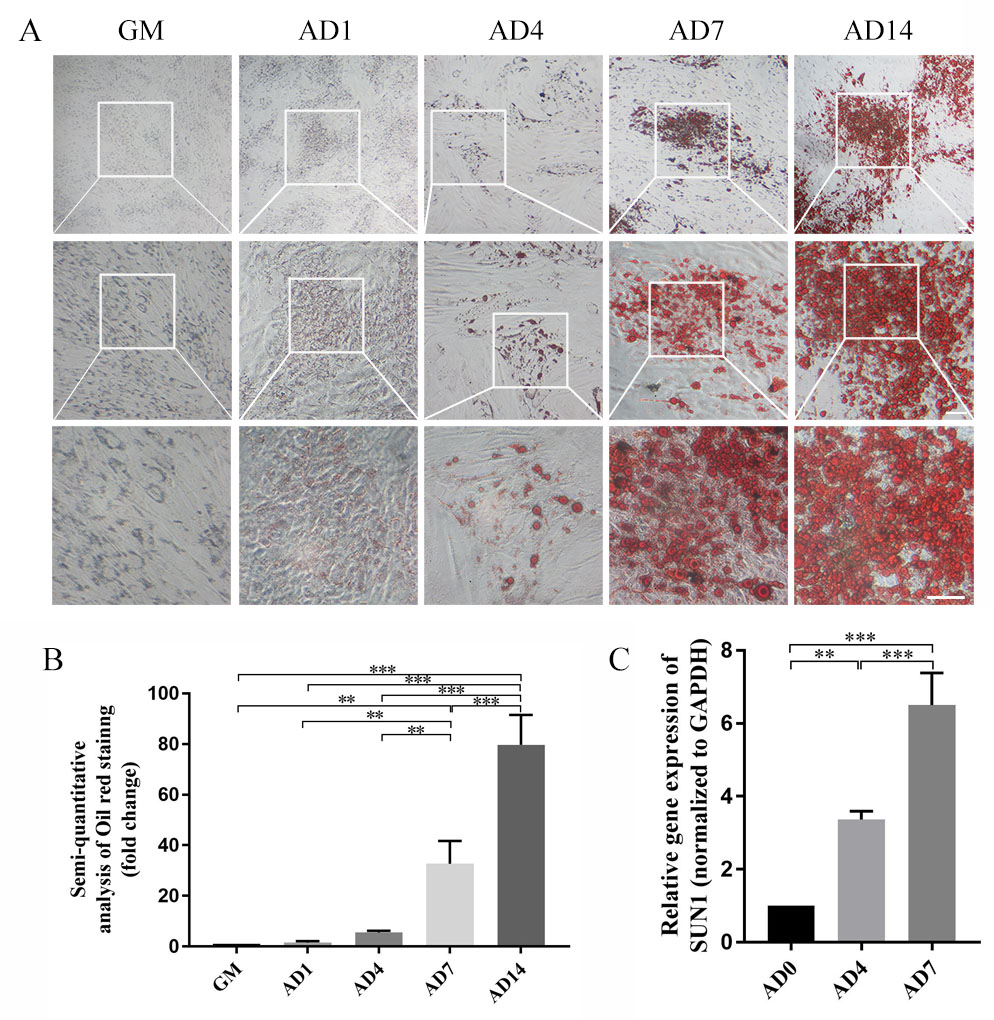
**

**Figure S2 Identification of adipogenic differentiation of hASCs. (A-B)** Representative images of oil red O staining and semi-quantitative analysis. **(C)** qRT-PCR assay analyzes the gene expression of SUN1. GM, growth medium; AD, adipogenic differentiation medium. AD1, AD4, AD7, and AD14 representing day 1, 4, 7, and 14 of adipogenic differentiation, respectively. Scale bar, 50 μm. Experiments were repeated at least three times (n ≥ 3). **P* < 0.05, ***P* < 0.01, ****P* < 0.001.


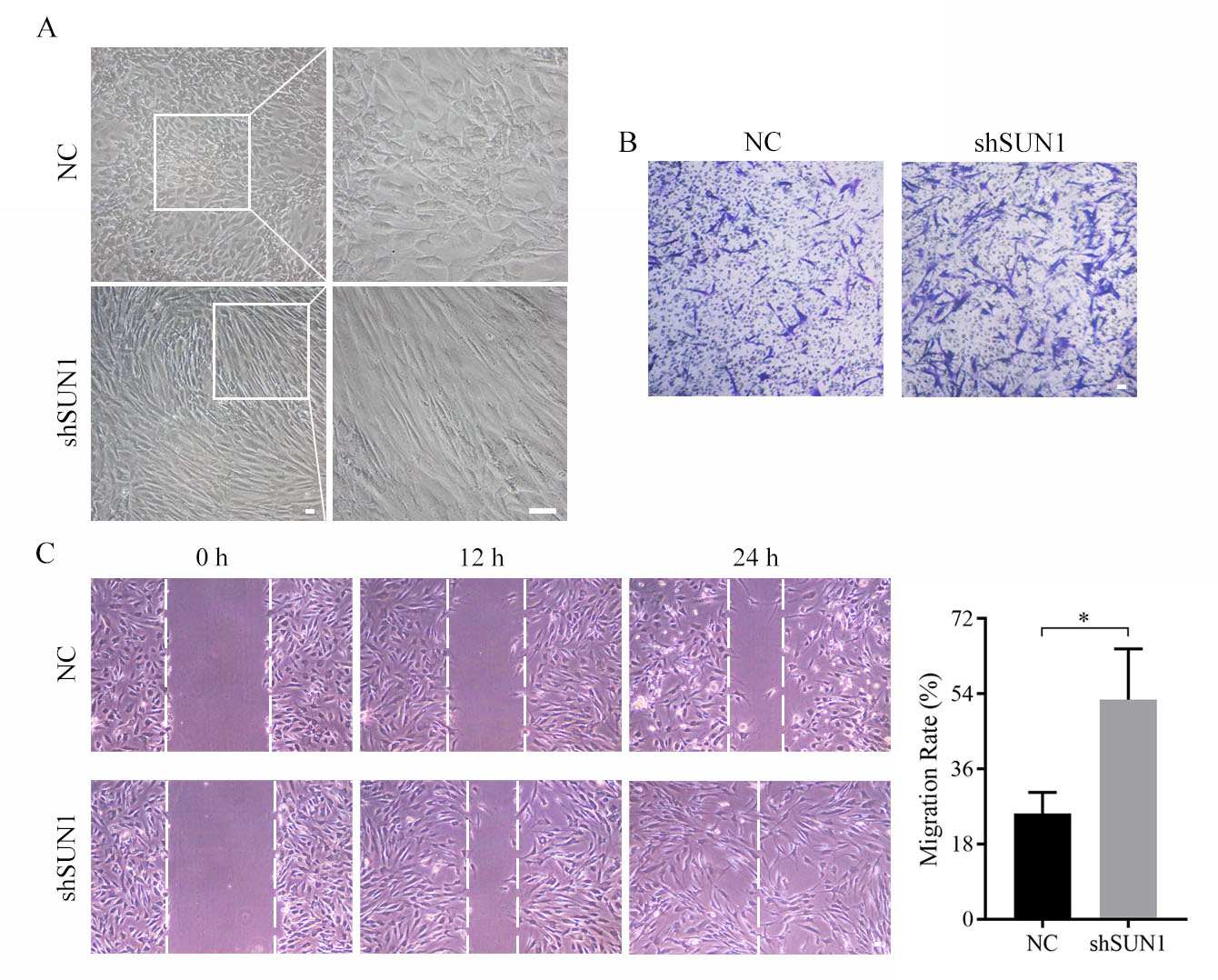


**Figure S3 Effect of SUN1 downregulation on hASCs performance.** **(A)** The microscopic observation images. Scale bar, 50 μm. **(B)** Transwell migration assay. Scale bar, 50 μm. **(C)** Wound Healing assay and quantitative analysis of wound healing area at 12 h. Scale bar, 50 μm. NC represented negative control cells. shSUN1, SUN1 down-regulated cells. Experiments were repeated at least three times (n ≥ 3). **P* < 0.05, ***P* < 0.01, ****P* < 0.001.


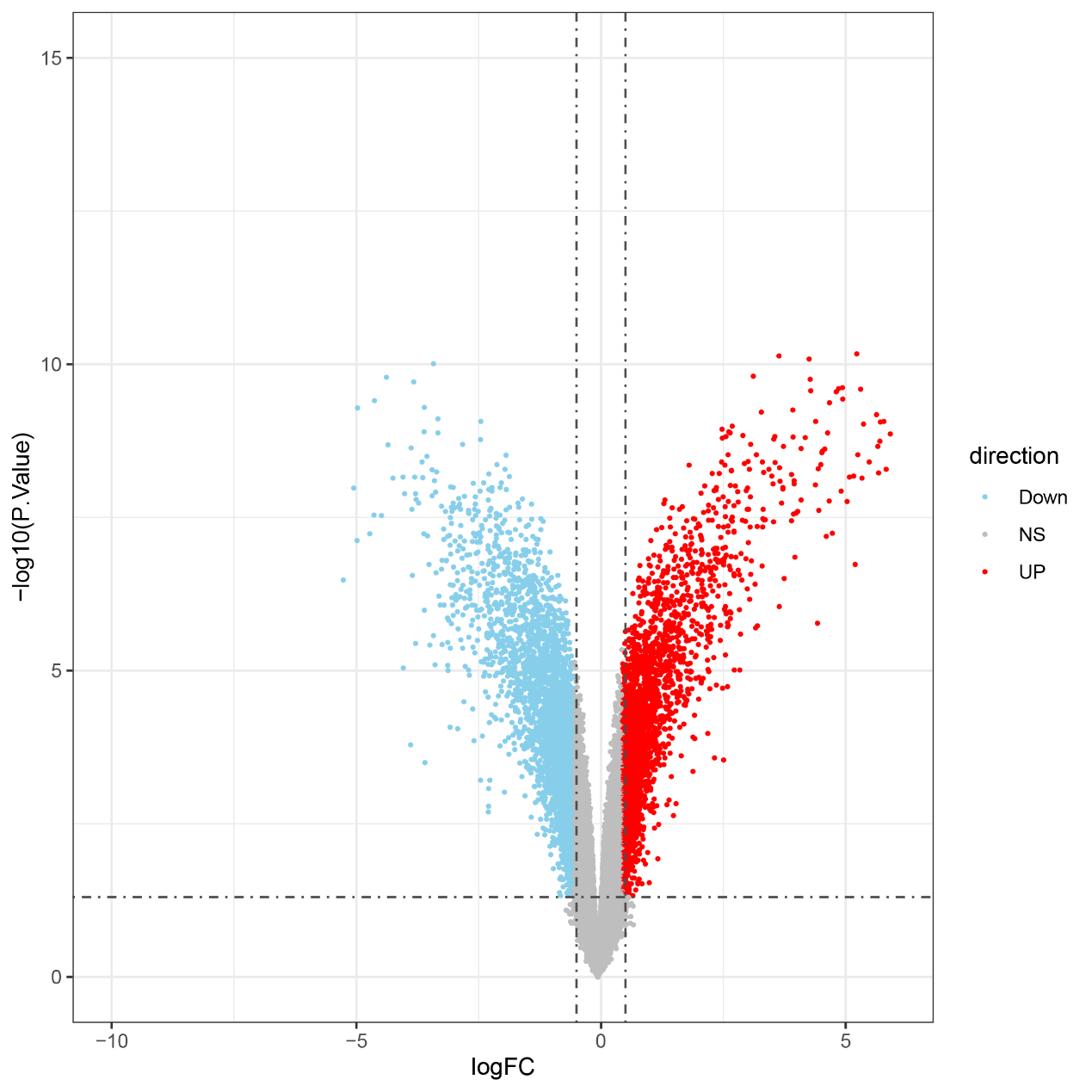


**Figure S4 Volcano plot of** **DEGs.** The volcano map shows the DEGs between osteogenic differentiation and lipogenic differentiation. Red points, significantly upregulated genes; blue points, significantly downregulated genes; gray points, no significant change genes. The X-axis shows the log2 fold change in gene expression, while the Y-axis shows the -log10 (*p*-value).
